# Association of Firearm Access, Use, and Victimization During Adolescence With Firearm Perpetration During Adulthood in a 16-Year Longitudinal Study of Youth Involved in the Juvenile Justice System

**DOI:** 10.1001/jamanetworkopen.2020.34208

**Published:** 2021-02-04

**Authors:** Linda A. Teplin, Nicholas S. Meyerson, Jessica A. Jakubowski, David A. Aaby, Nanzi Zheng, Karen M. Abram, Leah J. Welty

**Affiliations:** 1 Department of Psychiatry and Behavioral Sciences, Northwestern University Feinberg School of Medicine, Chicago, Illinois; 2 Department of Medicine, Infectious Disease, Northwestern University Feinberg School of Medicine, Chicago, Illinois; 3 Institute for Policy Research, Northwestern University, Evanston, Illinois; 4 Department of Sociology, Weinberg College of Arts & Sciences, Northwestern University, Evanston, Illinois; 5 School of Education and Public Policy, Northwestern University, Evanston, Illinois; 6 Department of Preventive Medicine, Northwestern University Feinberg School of Medicine, Chicago, Illinois

## Abstract

**Importance:**

Preventing firearm violence requires understanding its antecedents. Yet no comprehensive longitudinal study has examined how involvement with firearms during adolescence—use, access, and victimization (defined as threatened with a weapon or gunshot injury)—is associated with the perpetration of firearm violence in adulthood.

**Objective:**

To examine the association between firearm involvement during adolescence and subsequent firearm perpetration and ownership in adulthood among youth involved in the juvenile justice system.

**Design, Setting, and Participants:**

This cohort study analyzed interview responses of 1829 randomly selected participants as part of the Northwestern Juvenile Project, a longitudinal study of health needs and outcomes of youth sampled from a temporary juvenile detention center in a large US city. Youth aged 10 to 18 years were interviewed in detention from November 1995 through June 1998. Participants were reinterviewed up to 13 times over 16 years through February 2015, for a total of 17 776 interviews. The sample was stratified by sex, race/ethnicity, age, and legal status (juvenile or adult court). Data were analyzed from April 2017, when data preparation began, through November 2020.

**Exposures:**

Firearm involvement during adolescence: use (ie, threaten, shoot), access (ownership, ease of access, firearm in household, membership in gang that carries firearms), and victimization (gunshot injury, threatened with a weapon).

**Main Outcomes and Measures:**

Firearm involvement during adulthood: perpetration of firearm violence (ie, threatening with or using a firearm) and firearm ownership.

**Results:**

Among the 1829 participants, 1388 had a 16-year follow-up interview: 860 males, 528 females; 809 were African American, 203 were non-Hispanic White; 374 were Hispanic; and 2 were other race/ethnicity; median (interquartile range) age of 32 (30-32) years. Eighty-five percent of males and 63.2% of females were involved with firearms as adolescents. Compared with females, males had significantly higher odds of every type of involvement except having a firearm in the home. In adulthood, 41.3% of males and 10.5% of females perpetrated firearm violence. Adolescents who had been threatened with a weapon or injured by firearms had 3.1 (95% CI, 2.0-4.9) and 2.4 (95% CI, 1.2-4.9) times the odds of perpetrating violence during adulthood. Similar associations were found for firearm ownership.

**Conclusions and Relevance:**

Involvement with firearms during adolescence—including victimization—is a significant risk factor for firearm perpetration and ownership during adulthood.

## Key Points

**Question:**

Are youths involved in the juvenile justice system who use, have access to, or have been injured by a firearm or threatened with a weapon during adolescence more likely to perpetrate firearm violence and own firearms in adulthood?

**Findings:**

This cohort study of 1829 randomly selected youth newly detained in a temporary juvenile detention center found that 85% of males and 63% of females were involved with firearms as adolescents. Nearly all types of firearm involvement during adolescence were associated with increased odds of using and owning firearms during adulthood.

**Meaning:**

These findings suggest that firearm use, access, injury, and being threatened with a weapon during adolescence may be risk factors for firearm perpetration and ownership in adulthood.

## Teaser

This cohort study of youth interviewed while detained in a temporary juvenile detention center and reinterviewed up to 16 years later examines the association of adolescent exposure to firearms with firearm use and ownership as an adult.

## Introduction

Firearm-related violence is a serious public health problem in the US,^1^ responsible for nearly 200 000 homicides from 2003 to 2018.^2^ Despite declines in homicide rates over the past 3 decades,^3^ the proportion of homicides involving firearms is at an all-time high (72% in 2018).^4^ Although the public is justifiably horrified by the increasing frequency of mass shootings, the most common victims of interpersonal firearm violence in the US continue to be low-income urban populations.^1^ African American individuals—especially male young adults—disproportionately experience firearm violence.^1,5^

Preventing firearm violence requires understanding its antecedents. Prior research suggests that children who are involved with firearms are more likely to be involved with them as adults. Children who carried firearms,^6,7^ had peers who owned firearms for protection (vs for hunting or sport),^8^ and those exposed to firearm violence^9^ are more likely to carry firearms through their early and mid-20s. However, far fewer studies examine how involvement with firearms—use, access, and victimization (defined as threatened with a weapon or gunshot injury)—is associated with the perpetration of firearm violence.

To assess the literature, we searched PsycINFO, PubMed, Web of Science, Scopus, and Google Scholar for studies that met the following criteria: (1) assessed firearm perpetration as an outcome variable, (2) studied children sampled between ages 10 and 18 years, and (3) were published in a peer-reviewed journal since 1990.

Twelve studies met these criteria. (Literature table available from authors upon request.) Eight of these studies were cross-sectional,^10,11,12,13,14,15,16,17^ focusing only on the contemporaneous association between involvement with firearms (eg, number owned, reasons for carrying, how acquired) and firearm violence. Four studies were longitudinal. Bjerregaard and Lizotte^18^ studied 987 “high-risk youth” (ie, sampled from high-crime areas). Rowan et al^19^ studied a subsample of convicted juvenile offenders, all of whom had self-reported perpetration of firearm violence. Neither study, however, examined how involvement with firearms during adolescence was associated with perpetration of firearm violence in adulthood. Gonzales and McNiel^20^ and Pardini et al^21^ used data from Pathways to Desistance, a study of 1354 juvenile offenders (ages 14-18 years) who had been convicted of “serious” offenses (eg, felonies or weapons-based misdemeanors). Participants were followed for 7 years, through ages 21 to 25 years. Gonzales and McNiel^20^ found that firearm perpetration (defined as “shooting at someone”) was associated with firearm perpetration at later follow-ups. However, their other analyses of firearm involvement and firearm perpetration focused on contemporaneous relationships. Pardini et al^21^ examined predictors of firearm perpetration from 1 wave to the next (6 months to 1 year later) among males. Thus, neither study was able to address how involvement with firearms during adolescence (eg, ownership, ease of access, and victimization) was associated with firearm perpetration in adulthood. Moreover, the sample—convicted serious juvenile offenders—represents a small fraction of youth processed in the juvenile justice system.^22,23^

In this article, we present data from the Northwestern Juvenile Project, a 16-year prospective longitudinal study of health needs and outcomes of 1829 youth, up to median age 32 years, who had been arrested and detained in the juvenile justice system.^24,25,26^ This population is important to study for 2 reasons. First, victimization from^27,28^ and perpetration of^10,11,12,13,20^ firearm violence are common among youth in the juvenile justice system. Second, a relatively small proportion of perpetrators account for a large proportion of violent incidents.^29^ Studying youth in the juvenile justice system will provide needed data on a group that is at high risk for perpetration and vulnerable for victimization. To our knowledge, this is the first comprehensive longitudinal study examining how involvement with firearms during adolescence—use (ie, threaten, shoot), access (ownership, ease of access, firearm in household, membership in gang that carries firearms), and victimization (gunshot injury, threatened with a weapon)—is associated with subsequent use and ownership of firearms in adulthood.

## Methods

### Sample and Procedures

We recruited a stratified random sample of 1829 youth who were arrested, detained, and awaiting adjudication or disposition of their case at the Cook County Juvenile Temporary Detention Center (CCJTDC) in Chicago, Illinois, between November 20, 1995, and June 14, 1998. Participants signed either an assent form (if their age was <18 years old) or, for subsequent interviews, a consent form (if ≥18 years old). The institutional review boards of Northwestern University, the US Centers for Disease Control and Prevention, the Illinois Department of Child and Family Services, Cermak Health Services, the Illinois Department of Corrections, and the Federal Bureau of Prisons approved relevant study procedures. The Northwestern University institutional review board, Cermak Health Services, and the Illinois Department of Corrections also waived parental consent when participants were younger than 18 years, consistent with federal regulations regarding research with minimal risk.^30^ Additional detail is in the eAppendix in the Supplement and is published elsewhere.^24,26,31,32^ This study followed the Strengthening the Reporting of Observational Studies in Epidemiology (STROBE) reporting guideline.

Participants were sampled at intake. The CCJTDC is used for pretrial detention and for offenders sentenced for fewer than 30 days. To ensure adequate representation of key subgroups, we (1) stratified our sample by sex, race/ethnicity (ie, African American, non-Hispanic White, Hispanic, other), age (10-13 years or 14-18 years), and legal status (processed in juvenile or adult court); and (2) oversampled participants from underrepresented strata (eg, Hispanic young women). Final sampling fractions ranged from 0.018 to 0.689. Face-to-face structured interviews were conducted at the detention center in a private area, most within 2 days of intake.

We conducted follow-up interviews at approximately 3, 5, 6, 8, 12, 14, 15, and 16 years after the baseline interview (hereafter referred to as “after detention”) for the entire sample; subsamples were interviewed at 3.5, 4, 10, 11, and 13 years after detention. Participants were interviewed whether they lived in the community or in correctional facilities; 81.6% of participants still alive had an interview at year 16; 96% of participants had at least 1 interview during adulthood. The 16-year follow-up interviews were conducted from February 23, 2012, through February 12, 2015.

### Measures and Variables

#### Firearms: Access, Perpetration, and Victimization

At all follow-up interviews, we asked participants about firearm use (ie, ever, since last interview, and age at first use); access to firearms (current ownership, current firearm in household, current ease of obtaining a firearm, and membership in a gang that carries firearms ever and since last interview); victimization (gunshot injury ever and since last interview; threatened with a weapon ever and since last interview); and perpetration of firearm violence (firing a firearm or showing a firearm in a threatening manner ever and since last interview, and age at first perpetration). Persons who were incarcerated for 30 days before an interview were not asked about certain behaviors (eg, owning a firearm, easy access) because prisoners are not allowed to keep weapons. At the 16-year interview, we added retrospective questions about gunshot injury during adolescence for a subsample of participants. The sample sizes used for analyses of specific risk factors vary because of these contingencies (see eAppendix in the Supplement for additional information).

Information on deaths was obtained or verified during contacts with participants’ friends, family members, or other acquaintances by checking death records from state medical examiners’ offices, from online news sources, and by submitting our participants’ names to the National Death Index.

### Statistical Analysis

All analyses were conducted with Stata, version 15 (StataCorp) using its survey routines.^33^ StatTag version 6.0 was used to connect Stata output to manuscript text.^34^ We reported the prevalence of firearm involvement before age 18 years by sex and race/ethnicity (self-identified as African American, Hispanic, non-Hispanic White, or other). We used logistic regression to estimate demographic differences. To generate prevalence estimates and inferential statistics that reflect CCJTDC’s population, each participant was assigned a sampling weight to account for stratified sampling. As is standard procedure for analysis of stratified, weighted health surveys we (1) adjusted sampling weights for nonresponse to account for missing data,^35,36^ and (2) used Taylor series linearization to estimate standard errors.^36,37^ We used 2-sided tests with α = .05 to assess statistical significance.

#### Firearm Involvement in Childhood and Perpetration and Ownership in Adulthood

We used generalized linear mixed models (GLMMs)^38,39^ to examine associations between types of firearm involvement before age 18 years and firearm use or ownership through young adulthood (18 years up to median age 32 years). We chose GLMMs because they can be formulated to (1) model use/ownership throughout young adulthood; (2) account for a binomial outcome (ie, firearm use [yes/no]); and (3) allow for repeated measurements on participants. We used a binomial mixed model with a logit link to examine use or ownership of firearms at all follow-up interviews in young adulthood. We used all available interviews in young adulthood, an average of 6 interviews per person (range, 1-13 interviews). A participant-specific random intercept was included to account for repeated measurements on participants throughout adulthood. All GLMM models included covariates for sex, race/ethnicity, and age at detention (10-18 years). To account for how firearm use and ownership may change as a participant ages, we modeled time since detention using restricted cubic splines with 3 interior knots. We included an offset for time at risk (ie, days since the previous interview minus days incarcerated) because participants are prohibited from using or owning a firearm when incarcerated. Four participants who identified as other race/ethnicity were excluded.

#### Missing Data

To assess how attrition could affect generalizability, we used logistic regression to compare demographic characteristics of participants who received a 16-year interview with those who did not. Potential bias from demographic differences in attrition was adjusted for by weighting estimates by sampling strata.

## Results

The baseline sample included 1172 males and 657 females; 1005 participants were African American, 296 were non-Hispanic White, 524 were Hispanic, and 4 had other race/ethnicity; the mean (SD) age was 14.9 (1.4) years (Table 1). Sixteen years after baseline, 120 participants had died, 88 had withdrawn or refused participation, 220 could not be located, and 13 were interviewed out of range. Our analysis includes the remaining 1388 participants (860 males, 528 females; 809 were African American, 203 were non-Hispanic White; 374 were Hispanic; and 2 were other race/ethnicity) (Table 1). Compared with males, females were more likely to be retained 16 years after detention (odds ratio [OR], 1.48; 95% CI, 1.18-1.87). African American participants were more likely to be retained compared with non-Hispanic White participants (OR, 1.89; 95% CI, 1.41-2.53) and Hispanic participants (OR, 1.65; 95% CI, 1.29-2.11).

**Table 1.** Demographic Characteristics and Retention of the Original Sample Recruited From the Cook County Juvenile Temporary Detention Center Between 1995 and 1998^a^

Characteristic	Participants, No. (%)		
	Baseline (n = 1829)	Missing 16 y after baseline (n = 441)	Final sample 16 y after baseline (n = 1388)^b^
Race/ethnicity			
African American	1005 (54.9)	196 (44.4)	809 (58.3)
Non-Hispanic White	296 (16.2)	93 (21.1)	203 (14.6)
Hispanic	524 (28.6)	150 (34.0)	374 (26.9)
Other	4 (0.2)	2 (0.5)	2 (0.1)
Sex			
Male	1172 (64.1)	312 (70.7)	860 (62.0)
African American	575 (31.4)	128 (29.0)	447 (32.2)
Non-Hispanic White	207 (11.3)	63 (14.3)	144 (10.4)
Hispanic	387 (21.2)	119 (27.0)	268 (19.3)
Other	3 (0.2)	2 (0.5)	1 (0.1)
Female	657 (35.9)	129 (29.3)	528 (38.0)
African American	430 (23.5)	68 (15.4)	362 (26.1)
Non-Hispanic White	89 (4.9)	30 (6.8)	59 (4.3)
Hispanic	137 (7.5)	31 (7.0)	106 (7.6)
Other	1 (0.1)	0	1 (0.1)
Legal status at detention			
Processed in adult court	275 (15.0)	55 (12.5)	220 (15.9)
Processed in juvenile court	1554 (85.0)	386 (87.5)	1168 (84.1)
Age, y			
Mean (SD)	14.9 (1.4)	NR	32.0 (1.4)
Median (range)	15 (10-18)	NR	32 (26-36)
Nonresponse			
Died	NA	120 (27.2)	NA
Refused/withdrew	NA	88 (20.0)	NA
Skipped^c^	NA	220 (49.9)	NA
Out of range^d^	NA	13 (2.9)	NA
Interview type			
Community	NA	NA	930 (67.0)
Incarcerated	NA	NA	244 (17.6)
Phone	NA	NA	212 (15.3)
Placement^e^	NA	NA	1 (0.1)

Abbreviations: NA, not applicable; NR, not reported.

^a^ Percentages may not sum to 100% because of rounding error.

^b^ At the 16-year follow-up interview, a subsample (389 participants) received a module on weapon-related injury.

^c^ Participant was not located in time to be interviewed for the current wave.

^d^ These interviews were excluded because (1) they occurred at a time point that was too close to the preceding interview or (2) they occurred after the planned cutoff date.

^e^ Participant lived in group quarters other than a correctional facility.

### Firearm Involvement Prior to Age 18 Years

Eighty-five percent of males and 63.2% of females were involved with firearms as adolescents (before age 18 years). Table 2 shows prevalence estimates and sex differences in types of involvement. Approximately three-quarters of males reported “easy access” to a firearm (71.0%), “any use of a firearm” (74.2%), and/or that they had been “threatened with a weapon” (76.5%). The most common involvement among females was being “threatened with a weapon” (61.2%) and having “easy access” to a firearm (49.8%). Males had significantly higher odds than females of every type of involvement except “firearm in the home.” For example, males had 7.1 (95% CI, 5.3-9.4) times the odds of using a firearm, and 4.9 (95% CI, 2.1-11.9) times the odds of owning one. Nearly 10% of males (9.5%) and 3.0% of females had a gunshot injury before age 18 years.

**Table 2.** Sex Differences in the Prevalence of Firearm Involvement Before Age 18 Years^a^

Firearm involvement	Prevalence, % (SE)		Difference, OR (95% CI)^b^
	Male	Female	
Use, ownership, or access			
Easy access	71.0 (5.0)	49.8 (4.7)	2.5 (1.36 to 4.51)
Any use of a firearm	74.2 (2.2)	28.9 (1.8)	7.1 (5.30 to 9.43)
Age first used a firearm, mean (SE), y^c^	13.9 (0.1)	14.5 (0.1)	−0.7 (−1.04 to −0.28)
Firearm in home	17.3 (4.6)	9.5 (2.7)	2.0 (0.83 to 4.76)
Owned a firearm	28.6 (5.7)	7.5 (2.4)	4.9 (2.05 to 11.93)
Gang carried firearm	24.5 (4.1)	13.2 (2.8)	2.1 (1.12 to 4.08)
Victimization			
Threatened with a weapon	76.5 (2.7)	61.2 (2.2)	2.1 (1.46 to 2.90)
Gunshot injury	9.5 (2.1)	3.0 (1.1)	3.4 (1.42 to 8.01)

Abbreviation: OR, odds ratio.

^a^ Estimates are weighted to adjust for sampling design and nonresponse and to reflect the demographic characteristics of the Cook County Juvenile Temporary Detention Center.

^b^ Differences are reported as ORs with the exception of age first used a firearm, for which we report the mean difference.

^c^ Only among participants who reported any firearm use before age 18 years. Among the participants who reported any firearm use ever, the mean (SE) age at first firearm use was 14.0 (0.1) years among males and 15.1 (0.2) years among females.

Racial/ethnic differences are shown in the eTable in the Supplement. African American and Hispanic males were more likely to use firearms than non-Hispanic White males. A quarter of Hispanic males had been injured by firearms before age 18 years, significantly more than African American (6.9%) and non-Hispanic White males (5.2%). However, non-Hispanic White males were more likely than Hispanic males to be threatened with a weapon. Nearly 40% of Hispanic females had used a firearm, significantly more than African American or non-Hispanic White females. Among those who had used a firearm before age 18 years, non-Hispanic White females were 1.5 years younger when they first used firearms compared with African American females (13.3 and 14.8 years, respectively).

### Association Between Firearm Involvement in Adolescence and Firearm Perpetration and Ownership in Adulthood

#### Firearm Perpetration in Adulthood

In adulthood, 41.3% of males and 10.5% of females perpetrated firearm violence. Depending on the type of behavior during adolescence, between 44% and 67% of participants who had been involved with firearms before age 18 years perpetrated firearm violence as adults (Table 3). All but 1 type of involvement before age 18 years was associated with firearm perpetration in adulthood (Table 3; eFigure 1 in the Supplement). Notably, participants who had owned a firearm during adolescence had 9.0 (95% CI, 4.5-18.2; *P* < .001) times the odds of perpetrating firearm violence in adulthood. Victimization was also significant: adolescents who had been threatened with a weapon or injured by gunshot had 3.1 (95% CI, 2.0-4.9; *P* < .001) and 2.4 (95% CI, 1.2-4.9; *P* = .01) times the odds of perpetrating firearm violence in adulthood, respectively.

**Table 3.** Prevalence of Firearm Perpetration in Adulthood by Exposure to Firearms During Adolescence^a^^,^^b^

Adolescent risk factor	Prevalence, % (SE)		OR (95% CI)	*P* value
	Among exposed	Among unexposed		
Easy access	44.7 (6.9)	24.9 (7.2)	5.1 (2.45-10.74)	<.001
Gang carries firearms	62.8 (7.9)	31.3 (4.7)	1.7 (.91-3.16)	.10
Used a firearm	46.0 (2.9)	20.8 (3.5)	5.1 (3.59-7.31)	<.001
Firearm in household	60.3 (13.0)	35.4 (5.7)	3.6 (1.48-8.53)	.005
Owned a firearm	67.0 (11.1)	29.9 (5.6)	9.0 (4.48-18.23)	<.001
Threatened with weapon	43.8 (3.2)	20.6 (4.7)	3.1 (1.99-4.89)	<.001
Gunshot injury	48.4 (11.1)	34.0 (3.7)	2.4 (1.21-4.90)	.01

Abbreviation: OR, odds ratio.

^a^ Estimates are weighted to adjust for sampling design, nonresponse, and to reflect the demographic characteristics of the Cook County Juvenile Temporary Detention Center.

^b^ Prevalence estimates are for firearm perpetration at any time point during young adulthood. Odds ratios compare firearm perpetration throughout young adulthood between the exposed and unexposed groups.

#### Firearm Ownership in Adulthood

Firearm ownership was prevalent: 37.9% among males and 21.4% among females. Depending on the type of behavior during adolescence, between 34% and 64% of participants who had been involved with firearms before age 18 years owned a firearm as adults (Table 4). Table 4 and eFigure 2 in the Supplement present adjusted ORs for the association between type of involvement with firearms before age 18 years and firearm ownership in adulthood. Nearly every type of involvement during adolescence—including victimization—was associated with firearm ownership in adulthood. For example, adolescents who had been threatened with a weapon had 2.6 (95% CI, 1.7-4.2; *P* < .001) times the odds of owning a firearm as adults.

**Table 4.** Prevalence of Firearm Ownership in Adulthood by Exposure to Firearms During Adolescence^a^^,^^b^

Adolescent risk factor	Prevalence, % (SE)		OR (95% CI)	*P* value
	Among exposed	Among unexposed		
Easy access	35.2 (6.5)	25.1 (7.2)	5.5 (2.55-11.84)	<.001
Gang carries firearms	60.8 (8.0)	22.4 (3.9)	3.7 (1.86-7.41)	<.001
Used a firearm	37.8 (2.8)	27.9 (3.9)	6.2 (4.31-9.05)	<.001
Firearm in household	54.5 (13.3)	29.3 (5.2)	6.4 (2.47-16.65)	<.001
Owned a firearm	64.2 (11.2)	22.7 (4.6)	16.9 (7.89-36.29)	<.001
Threatened with weapon	39.6 (3.2)	17.6 (3.8)	2.6 (1.68-4.15)	<.001
Gunshot injury	34.2 (10.1)	31.4 (3.6)	1.9 (.81-4.50)	.14

Abbreviation: OR, odds ratio.

^a^ Estimates are weighted to adjust for sampling design, nonresponse, and to reflect the demographic characteristics of the Cook County Juvenile Temporary Detention Center.

^b^ Prevalence estimates are for firearm ownership at any time point during young adulthood. Odds ratios compare firearm perpetration throughout young adulthood between the exposed and unexposed groups.

## Discussion

This prospective study is the first to our knowledge that finds that firearm involvement during adolescence—including victimization—is related to firearm perpetration in adulthood. In our sample of youth involved in the juvenile justice system, nearly all aspects of firearm involvement (eg, owning a firearm; having easy access; using a firearm; having a firearm in the household; having been threatened with a weapon; and having been injured by firearm) during adolescence were associated with increased odds of perpetrating firearm violence in adulthood. Most of these variables were also associated with firearm ownership in adulthood. For example, participants who were threatened with a weapon before age 18 years had 3.1 times the odds of perpetrating firearm violence in adulthood and 2.6 times the odds of owning a firearm. Our findings extend those of 2 community studies, which found that carrying a weapon to school and having access to firearms during adolescence were associated with gun carrying^6^ and firearm ownership^40,41^ up to age 26 years, respectively.

Our findings prevailed despite 2 countervailing trends. First, delinquent^42,43,44^ and violent behavior^45,46^ tends to decrease with age. Second, at the population level, firearm violence decreased during the period of our study (ie, the late 1990s to the 2010s), both locally^47^ and nationally.^48^

Our findings are of concern because firearm involvement during adolescence was common in our sample, especially among males. For example, approximately three-quarters of males reported having easy access to a firearm, having used a firearm, and/or having been threatened with a weapon. Firearm involvement among females during adolescence was slightly lower, but nevertheless substantial. For example, approximately 50% of females reported having easy access to a firearm and more than 60% reported having been threatened with a weapon. The observed sex and racial/ethnic differences in firearm involvement are similar to findings from prior studies of firearm victimization,^48^ perpetration,^49^ and mortality.^48,50^ In a 2014 analysis of our sample,^28^ we found that 16 years after detention, males had more than 7 times the risk of dying from homicide compared with females; most died from firearm injuries. Compared with non-Hispanic White young adults, African American young adults had more than 4 times the risk of death due to homicide, and Hispanic young adults had nearly 3 times the risk.^28^

What are the possible causal mechanisms between firearm involvement in adolescence and firearm perpetration and ownership in adulthood? Over time, risk factors accrue and interact, leading to a continuity of disadvantage.^51,52^ For example, youth who are victimized at school may perform poorly,^53,54^ become truant,^55^ and eventually drop out,^56^ limiting their employment opportunities.^57,58^ Youth may then become involved with gangs and the drug economy, increasing exposure to high-risk situations as they age.^8,10,18,59,60^ Finally, exposure to violence—which may occur in the home, among peers, or in the streets—may precipitate carrying firearms for protection.^9,61,62^

### Future Research

Future research on this topic could take several directions. One avenue would be to investigate variables that reduce the likelihood of firearm violence. Many studies investigate risk factors^13,15,16,21,63,64,65,66,67,68,69,70^; far fewer studies examine variables that reduce firearm involvement.^21,65,71^ Knowledge of modifiable protective factors will provide the empirical basis for effective interventions.

Another avenue for further study would be to examine intergenerational patterns of firearm involvement. Vulnerability to risk,^72^ resiliency,^73^ and associated outcomes^74,75^ have an intergenerational component. Future studies should examine how parents’ involvement with firearms during their own adolescence—including victimization and perpetration—influences their children’s risk.

Future work should also focus on Hispanic youth. Our findings highlight Hispanic participants’ elevated risk for firearm involvement, similar to racial/ethnic differences found in the general population. Firearm-related homicide rates among Hispanic Americans, now the largest minority group in the US, are more than triple that of non-Hispanic White individuals: 4.6 per 100 000 compared with 1.5 per 100 000.^76^ Yet far less is known about the Hispanic population and firearm involvement. Criminal justice statistics often record Hispanic ethnicity unreliably^77^ or not at all.^78^ Many empirical studies have a Hispanic population that is too small to analyze as a separate category.

### Implications for Public Health

The findings of this study suggest a need to expand prevention and intervention programs for individuals and communities. Programs that target high-risk youth like our participants—in addition to targeting the neighborhoods where they live—will have the greatest impact. One example of a promising intervention is Cure Violence, which uses community outreach workers to interrupt the transmission of violence by focusing on youth who are involved with gangs or have been victims of shootings.^79,80^ Neighborhoods also matter: interpersonal firearm violence is concentrated in urban communities characterized by poverty and inordinate housing vacancies,^81,82,83^ conditions that increase access to illegal firearms.^84^ Nearly half of juvenile offenders who have access to firearms obtain them from outside the home.^85^ They may find them, steal them, borrow them from a friend, or rent them from their gang.^85,86^ We recommend the expansion of blight remediation programs that focus on neglected communities and eliminate sites used to store and sell illegal firearms.^87^ Broad implementation of programs targeting individuals and communities could curtail firearm involvement among adolescents and reduce shootings,^88,89,90,91,92,93^ firearm injuries,^90,94^ and firearm deaths^80,87,88,89,94,95,96,97^ in the neighborhoods where they are most likely to occur.

Policy makers should also consider training pediatricians to educate parents on safe storage of firearms. One in 3 children live in a home where firearms are present.^98^ In those households, nearly three-quarters of firearms are not stored safely.^98,99^ Pediatricians can counsel parents that ammunition should be removed and locked up separately,^100^ and that firearms must be stored using cable locks, lockboxes, gun safes, or trigger locks.^101,102^ Weighed against the risk of injury and death, the cost is negligible: $25 for a pistol-sized lock box and $130 for a full-size gun safe.^103^ Pediatricians can encourage indigent parents to participate in safe storage programs, such as Project ChildSafe,^104^ which provides free gun-safety kits.^103^ Implementing safe storage practices may prevent up to 32% of firearm deaths among youth.^105^

### Limitations

This study had several limitations. Our data are subject to the limitations of self-report. We did not control for socioeconomic status because nearly all youth who enter detention are from lower-income households. Although retention rates were high, participants lost to follow-up may have biased the sample. The analysis omitted 39 participants who died during adolescence (33 of whom died from firearm homicide). Generalizability is limited to a high-risk population: economically disadvantaged urban youth who have been arrested and detained. It was not feasible to study more than 1 jurisdiction. Finally, our findings may have underestimated the strength of the association between firearm involvement during adolescence and perpetration in adulthood because (1) some participants were reinterviewed in correctional facilities, which do not allow firearms; and (2) participants who were convicted felons, on probation, or on parole are prohibited from possessing or using firearms. Despite these limitations, our findings have implications for future research and public health.

## Conclusions

In our sample of youth involved in the juvenile justice system, nearly all aspects of firearm involvement during adolescence (eg, owning a firearm; having easy access to a firearm; using a firearm; having a firearm in the household; having been threatened with a weapon; and having been injured by a firearm) were associated with increased odds of perpetrating firearm violence and owning a firearm in adulthood. To reduce firearm injury and death, we advocate for the recommendations of the American Medical Association and the American Academy of Pediatrics, among them to increase funding for firearm violence prevention research; pass laws that impose criminal liability on adults who negligently leave firearms accessible to children; enhance children’s access to mental health services to address the consequences of their exposure to violence; oppose federal legislation that permits concealed carry reciprocity across state lines; limit ammunition magazines to 10 rounds; and reinstate the ban on military-style assault weapons.^100,106,107^ The health community is in a unique position to leverage its influence with local, state, and federal governments to implement reform.

## Notes

Supplement. **eAppendix.** Expanded Notes on Study Methods **eTable.** Firearm Involvement Prior to Age 18: Racial/Ethnic Differences for Male and Female Adolescents **eFigure 1.** Association Between Firearm Involvement During Adolescence and Firearm Perpetration in Adulthood **eFigure 2.** Association Between Firearm Involvement During Adolescence and Firearm Ownership in Adulthood **eReferences**
