# Supplementary material for: Association of Firearm Access, Use, and Victimization During Adolescence With Firearm Perpetration During Adulthood in a 16-Year Longitudinal Study of Youth Involved in the Juvenile Justice System
Source: JAMA Netw Open. 2021 Feb 4;4(2):e2034208. doi: 10.1001/jamanetworkopen.2020.34208 (PMC7862991; doi:10.1001/jamanetworkopen.2020.34208)
Supplement: Supplement. — eAppendix. Expanded Notes on Study Methods eTable. Firearm Involvement Prior to Age 18: Racial/Ethnic Differences for Male and Female Adolescents eFigure 1. Association Between Firearm Involvement During Adolescence and Firearm Perpetration in Adulthood eFigure 2. Association Between Firearm Involvement During Adolescence and Firearm Ownership in Adulthood eReferences [file jamanetwopen-e2034208-s001.pdf]

Supplemental Online Content

Teplin LA, Meyerson NS, Jakubowski JA, et al. Association of firearm access, use, and victimization during adolescence with firearm perpetration during adulthood in a 16-year longitudinal study of youth involved in the juvenile justice system. *JAMA Netw Open*. 2021;4(2):e2034208. doi:10.1001/jamanetworkopen.2020.34208

eAppendix. Expanded Notes on Study Methods

eTable 1. Firearm Involvement Prior to Age 18: Racial/Ethnic Differences for Male and Female Adolescents

eFigure 1. Association Between Firearm Involvement During Adolescence and Firearm Perpetration in Adulthood

eFigure 2. Association Between Firearm Involvement During Adolescence and Firearm Ownership in Adulthood

eReferences

This supplemental material has been provided by the authors to give readers additional information about their work.

© 2021 Teplin LA et al. *JAMA Network Open.*

eMethods. Expanded Notes on Study Methods

Background

Characteristics of the Cook County Juvenile Temporary Detention Center (CCJTDC)

Consistent with juvenile detainees nationwide,[1] nearly 90% of detainees at CCJTDC were male; most were racial/ethnic minority youth.

Sampling and Procedures

Participants were 1,829 male and female youth, 10 to 18 years old, randomly sampled from intake into the CCJTDC from November 20, 1995, through June 14, 1998. The sampling was stratified by sex, race/ethnicity (African American, non-Hispanic white, Hispanic, and "other" race/ethnicity), age (10-13 years or ≥14 years), and legal status (processed in juvenile or adult court) to obtain enough participants to examine key subgroups (e.g., females, Hispanics, younger persons). There were 13 strata, as listed below. There were too few female detainees of each race/ethnicity and detainees identified as "other" race/ethnicity to further stratify these groups. Detainees aged 10 to 13 years were not stratified by legal status because they were generally too young to be considered for transfer to adult court. We chose the sample size to allow sufficient power to detect prevalence of rare disorders across key subgroups (e.g. Hispanic males). Actual sampling was based on these estimates as well as feasibility. For example, as at urban detention facilities nationwide, few females or younger detainees (10-13 years of age) are processed as adults.

Strata:

- African American females
- Non-Hispanic white females
- Hispanic females
- African American males, aged 10-13 years
- Non-Hispanic white males, aged 10-13 years
- Hispanic males, aged 10-13 years
- African American males, 14 years or older and processed as adult transfer
- Non-Hispanic white males, 14 years or older and processed as adult transfer
- Hispanic males, 14 years or older and processed as adult transfer
- African American males, 14 years or older and processed as a juvenile
- Non-Hispanic white males, 14 years or older and processed as a juvenile
- Hispanic white males, 14 years or older and processed as a juvenile
- Other race/ethnicity

A study liaison was scheduled to work every day (including weekends) throughout the study. Each day, the liaison randomly selected potential participants within strata. Detainees were classified in strata using information listed in the intake log. The liaison sampled from the strata in a preset order. If no participants were available for a stratum, the liaison sampled from the next stratum. If multiple detainees were available for a stratum, the liaison used a random number table and the last digit of the CCJTDC ID number to randomly sample potential participants from within the stratum.[2] The final sampling fractions for the strata ranged from 0.018 to 0.689.

© 2021 Teplin LA et al. *JAMA Network Open.*

All detainees who were awaiting the adjudication or disposition of their case were eligible to participate in the study. Among these, 2275 detainees were randomly selected; 4.2% (34 youth and 62 parents or guardians) refused to participate. There were no significant differences in refusal by sex, race/ethnicity, or age. Twenty-seven youth left the detention center before an interview could be scheduled; 312 left CCJTDC while we attempted to locate their caretakers for consent. Eleven others were excluded from the sample because they were unable to complete the interview. The final sample size was 1829: 1172 males, 657 females; 1005 African Americans, 296 non-Hispanic whites, 524 Hispanics, 4 "other" race/ethnicity; age range, 10 to 18 years (mean, 14.9 years; median, 15 years) (see **Table 1**).

The most common offenses were violent crimes (55.4%), property crimes (43.5%), violation of probation or parole (36.4%), drug crimes (30.7%), and weapons crimes (13.5%). (Numbers sum to >100% because participants could be arrested for more than 1 charge.)

Face-to-face structured interviews were conducted at the detention center in a private area, most within 2 days of intake. Participants were paid $25 for the 2- to 3-hour baseline interview.

We re-interviewed participants irrespective of where they lived: in the community (65%-69% of interviews); at correctional facilities (15%-29% of interviews); by telephone if they lived more than 2 hours away (4%-16% of interviews); or in a placement, such as a group home (<2% of interviews.) Participants were paid $50 for the 3- through6-year follow-up interviews; $75 for the 8- through 13-year follow-up interviews; and $100 for the 14- through 16-year follow-up interviews, each lasting approximately 3 to 4 hours.

Youth Processed in Juvenile or Adult Court

Although most juvenile offenders are processed in juvenile court, all 50 states and the District of Columbia have legal mechanisms to try juveniles as adults in criminal court.[3,4] Transfers to adult criminal court typically result from: (1) judicial waiver on a case-by-case basis,[5-7] (2) automatic transfers based on the type of offense, criminal history, and age of the detainee,[5] and (3) prosecutorial direct-file mechanisms that allow prosecutors to determine when to file certain juvenile cases directly in adult criminal court.[5] The increased availability of legal mechanisms to process juveniles in adult criminal court is largely responsible for the 366% increase between 1983 and 1998 in the number of juveniles held in adult jails.[8] As of 2004, about 7% of the approximately 2 million arrests of youths eligible for processing in the juvenile justice system were cases in which the youth was transferred directly to adult criminal court.[1,9]

Procedures for Obtaining Parental Consent for Minor Youth for Baseline and Follow-up Interviews

For all interviews, participants signed either an assent form (if they were <18 years) or a consent form (if they were ≥18 years). The Northwestern University Institutional Review Board and the Centers for Disease Control and Prevention Institutional Review Board approved all study procedures and waived parental consent for persons younger than 18 years, consistent with federal regulations regarding research with minimal risk.[10] We nevertheless attempted to contact parents of minors to obtain their consent and to provide them with information on the study and used an independent participant advocate to represent the minors' interests.[10]

Baseline: Study liaisons tried to reach detainees' parents or guardians in 2 ways: first, they attempted to call them by telephone at least 3 times over 2 days. Second, they tried to obtain consent from the parents or guardians in person during visiting hours. A participant advocate acted on the child's behalf if the parents or guardians were not reachable. In the absence of a parent or guardian, the participant advocate protects the interests of the youth and determines that they are consenting voluntarily, understand the research procedure, and are not being coerced to participate. Consistent with federal regulations, we excluded detainees who did not wish to participate, even if their parents or guardians consented.[10,11]

Follow-up: Two weeks before a follow-up interview was due, a liaison telephoned the parent or guardian of minors to obtain their consent. If they provided consent, the liaison then contacted the youth to obtain assent and schedule their interview. The Illinois Department of Child and Family Services allowed us to recontact and interview participants who were under their guardianship, provided that we received assent from the youth. As with baseline interviews, we excluded detainees who did not wish to participate, even if their parents or guardians consented. Also as with baseline interviews, minors could still participate even if we could not reach their parent or guardian. If we could not reach them after one week and at least 5 attempts, we initiated the participant advocate system described earlier. In these cases, the liaison contacted the participant directly to request his or her assent. If we could not reach the participant by phone, an interviewer traveled to his or her location.[10,11]

Clinical Research Interviewers

For baseline and follow-up interviews, female participants were interviewed by female interviewers. Most interviewers had graduate degrees in psychology or an associated field and had experience interviewing at-risk youth; one-third were fluent in Spanish. All interviewers were trained for at least 1 month. Follow-up interviews were longer than baseline interviews because, at the request of our funding agencies, we added additional variables.

Measures and Variables

Variables on firearm perpetration were adapted from similar questions about delinquent behavior from the Denver Youth Survey.[12] We also developed additional questions on the onset and characteristics of firearm involvement for this study. At each follow-up interview, we asked participants about firearm use (recent use, age of first use); access to firearms (current ownership, firearm in the household, ease of obtaining a firearm, membership in a gang that carries firearms); victimization (gunshot injury; threatened with a weapon); and perpetration of firearm violence (firing a firearm or showing a firearm in a threatening manner: age at first perpetration, perpetration since last interview). At the 16-year interview, we added retrospective questions about gunshot injury during adolescence for a subsample of participants. Therefore, subsamples used for analyses of specific risk factors vary. Information on firearm-related deaths (homicide, suicide, or accident) were obtained or verified with official records from state medical examiners' offices.

Incarceration

Incarceration variables are based on data from official records. We obtained intake and exit dates for correctional stays from the Illinois Department of Corrections adult and youth divisions, the Cook County Department of Corrections, and the Clerk of the Court of Cook

County (for stays in the CCJTDC). Because it was not feasible to collect records for those in federal prisons, out-of-state prisons, and detention facilities outside of Cook County, dates for stays in these facilities are based on self-report (<2.74% of stays).

We matched official records to participants using names, known aliases, race/ethnicity, birth dates, and institutional ID numbers. Throughout the course of the study, we routinely updated identifying information each time a participant was contacted or interviewed. We also routinely updated institutional IDs every time a participant was interviewed in a correctional setting or discovered to be incarcerated through multiple mailings sent to participants each year of the study.

We generated the following variables at each follow-up interview: length of time incarcerated in the past year (days), an indicator variable for having been incarcerated during the entire past year (yes/no), and an indicator variable for living in the community during the entire past year (yes/no).

Measuring Participants during Adolescence and Adulthood

We conducted follow-up interviews at approximately 3, 5, 6, 8, 12, 14, 15, and 16 years after the baseline interview for the entire sample; subsamples were interviewed at 3.5, 4, 10, 11, and 13 years after the baseline interview. The table below lists the median age and age range of participants at each interview. Participants were followed an average of 14.8 years each, for a total of 27,023 years. (Information on deaths and dropouts are presented in **Table 1.**)

	**Interview (Years After Detention)**													
	**B**	**3**	**3.5**	**4**	**5**	**6**	**8**	**10**	**11**	**12**	**13**	**14**	**15**	**16**
**Median Age**	15	18	19	20	20	22	24	25	26	27	28	29	30	32
**Age Range**	10-18	13-25	13-26	14-25	14-26	16-27	18-30	20-29	22-30	22-32	23-32	24-33	25-34	26-36

Note. B = Baseline

Adolescence: All interviews that took place before a participant turned age 18 were used to determine their firearm involvement prior to age 18. Participants had between 1-6 interviews during adolescence. Approximately three-quarters of participants had one interview during adolescence; 28% of participants were interviewed more than once during adolescence. The proportion of participants who were younger than 18 years at each follow-up interview was: 29% (3 years after baseline); 24% (3.5 years after baseline); 15% (4 years after baseline); 7% (5 years after baseline); 1% (6 years after baseline). All participants were age 18 years or older at the 8-year follow-up interview.

Adulthood: All interviews that took place after a participant turned 18 were used to determine their firearm involvement throughout adulthood. Participants had between 1-13 interviews during adulthood; 94% of participants had more than 1 interview during adulthood. The majority of participants were 18 or older 3 years after baseline (median age at interview, 18 years old).

GLMMs Models for Association between Firearm Involvement in Childhood and Perpetration and Ownership in Adulthood

We used all available interviews, an average of 6 interviews per person (range, 1 -13 interviews). Ownership and use of firearms at each follow-up interview in young adulthood were modeled as binomials with a logit link function. All GLMM models included covariates for: sex, race/ethnicity, and age at detention (10-18 years). A participant-specific random slope was included to account for repeated measurements on participants throughout adulthood. In our exploratory analyses, we found evidence that perpetration and ownership changed non-linearly over time. We therefore modeled time since detention using restricted cubic splines with 3 interior knots (5 total knots). Knots were placed at equally spaced percentiles, following Harrell 2001[13] and as implemented in Stata 15. We chose 5 total knots because 3 to 5 total knots are generally considered adequate.[14,15] We included an offset for time at risk (days since the previous interview minus days incarcerated) because participants are prohibited from using or owning a gun when incarcerated. Four participants who identified as "other" race/ethnicity were excluded.

Item Missing
The N's for variables describing firearm use as an adolescent (prior to age 18) vary for several reasons: (1) most questions about firearm use were added at the first follow-up interview, at which time some participants were already over age 18 years; (2) participants who were incarcerated for 30 days prior to an interview were not asked about certain firearm-related behaviors because prisoners cannot keep weapons; and (3) retrospective questions about gunshot injury during adolescence were administered to a subsample (n = 389) of participants at the 16-year follow-up interview. The table below shows the sample sizes for each variable describing firearm use as an adolescent.

**Samples sizes for variables describing firearm use as an adolescent (prior to age 18).**	
**Variables**	**Sample**
Used a firearm;<br>Age when first used a firearm	Participants who received the first follow-up interview (n = 1741)
Gang carries firearms	Participants who were < 18 years old at the first follow-up interview (n = 506)
Owned a firearm;<br>Firearm in household;<br>Easy access	Participants who were < 18 years old at the first follow-up interview and were not incarcerated the entire 30 days prior to that interview (n =391)
Gunshot injury	Participants who were < 18 years old at the first follow-up interview OR were part of the n = 389 subsample at the 13th follow-up interview, 16 years after baseline. (n = 772)
Threatened with a weapon	Participants who received the PTSD module at baseline (13 months after the study began, when it became available) or who were < 18 at the first follow-up interview. (n = 1326)

Attrition
To assess the effect of attrition on generalizability, we compared demographic characteristics of participants who received a 16-year follow-up interview with those who did not (which included 118 participants who died before the 16-year follow-up). Females were more likely to be retained 16 years after detention compared with males (OR, 1.48; 95% CI, 1.17-1.87). African Americans were more likely to be retained 16 years after detention compared with

non-Hispanic whites (OR, 1.86; 95% CI, 1.39-2.49) and Hispanics (OR, 1.61; 95% CI, 1.26-2.06). Potential bias from demographic differences in attrition was adjusted by weighting the statistical analyses by sampling strata.

© 2021 Teplin LA et al. *JAMA Network Open.*

eTable 1. Firearm Involvement Prior to Age 18: Racial/Ethnic Differences for Male and Female Adolescents[a]							
**Firearm Involvement**	**African Americans**		**Non-Hispanic whites**		**Hispanics**		**Racial/Ethnic Differences[b]**
	**Males**						
Use, ownership, or access							
Easy access, % (se)	71.0	(6.1)	60.0	(9.0)	74.8	(7.4)	
Any use of a firearm, % (se)	73.3	(2.8)	62.7	(3.6)	82.2	(2.9)	H>AA; H>W; AA>W
Age 1st used a firearm, mean (se), yrs[c]	13.9	(0.2)	13.8	(0.2)	13.8	(0.2)	
Firearm in home, % (se)	19.1	(5.6)	12.4	(6.2)	9.1	(4.6)	
Owned a firearm, % (se)	31.3	(7.0)	14.2	(6.4)	18.9	(6.2)	
Gang carried firearm, % (se)	23.5	(4.9)	21.3	(7.3)	31.9	(6.7)	
Victimization							
Threatened with a weapon, % (se)	77.5	(3.2)	84.3	(3.1)	69.2	(5.6)	W>H
Gunshot injury, % (se)	6.9	(2.3)	5.2	(2.7)	23.9	(6.3)	H>AA; H>W
	**Females**						
Use, ownership, or access							
Easy access	50.6	(5.4)	53.8	(13.9)	42.3	(11.5)	
Any use of a firearm	27.3	(2.2)	22.4	(4.6)	39.7	(4.4)	H>AA; H>W
Age 1st used a firearm, mean (se), yrs[c]	14.8	(0.2)	13.3	(0.5)	14.2	(0.3)	AA>W
Firearm in home	8.3	(3.0)	14.3	(9.4)	12.8	(7.0)	
Owned a firearm	8.2	(3.0)	14.3	(9.4)	0.0	(0.0)	
Gang carried firearm	13.5	(3.4)	6.7	(6.5)	14.8	(6.2)	
Victimization							
Threatened with a weapon	60.2	(2.7)	56.1	(6.4)	68.6	(4.7)	
Gunshot injury	2.9	(1.3)	3.1	(3.1)	3.4	(2.4)	

Abbreviations: SE, Standard Error;

[a] Estimates are weighted to adjust for sampling design, nonresponse, and to reflect the demographic characteristics of the Cook County Juvenile Temporary Detention Center.

[b] Only statistically significant ($p < 0.05$) differences are indicated.

Males: Compared with non-Hispanic whites, Hispanics had 2.7 times the odds of using a firearm (95% CI, 1.68-4.50) and African Americans had 1.6 times the odds (95% CI, 1.09-2.47). Hispanics also had 1.7 times the odds of using a firearm compared with African Americans (95% CI, 1.04-2.71). Compared with Hispanics, non-Hispanic whites had 2.4 times the odds of being threatened with a weapon (95% CI 1.21-4.76). Hispanics had 4.2 times the odds of having a gunshot injury compared with African Americans (95% CI 1.60-11.14) and 5.8 times the odds compared with non-Hispanic whites (95% CI 1.59-20.99).

Females: Hispanics had 1.7 times the odds of using of a firearm compared with African Americans (95% CI 1.15-2.66) and 2.3 times odds compared with non-Hispanic whites (95% CI 1.21-4.26). African Americans were 1.5 years older than whites at age of first firearm use (95% CI 0.34-2.60).

[c] Only among participants who reported any firearm use prior to age 18.

© 2021 Teplin LA et al. *JAMA Network Open.*

eFigure 1. Association between Firearm Involvement during Adolescence and Firearm Perpetration in Adulthood

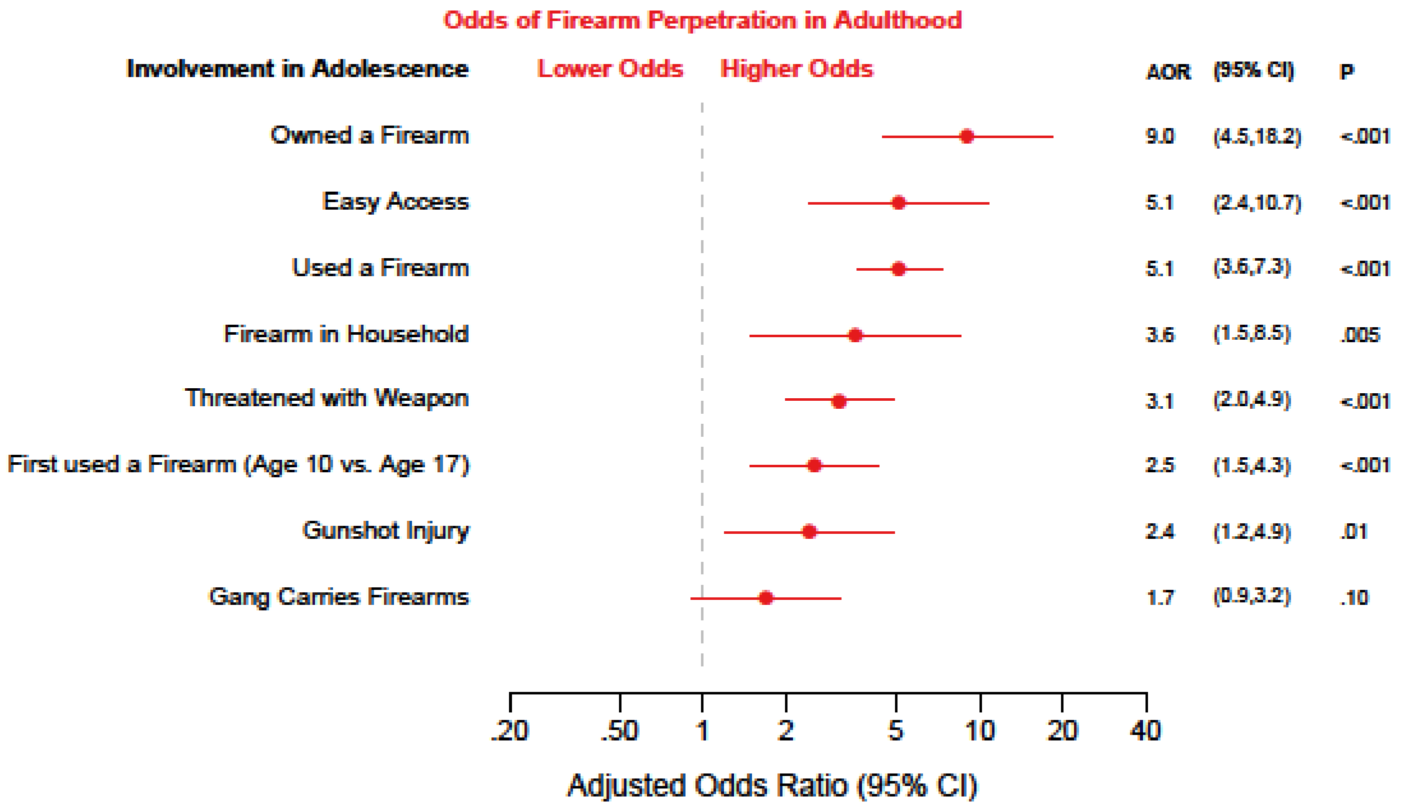

© 2021 Teplin LA et al. *JAMA Network Open.*

eFigure 2. Association between Firearm Involvement during Adolescence and Firearm Ownership in Adulthood

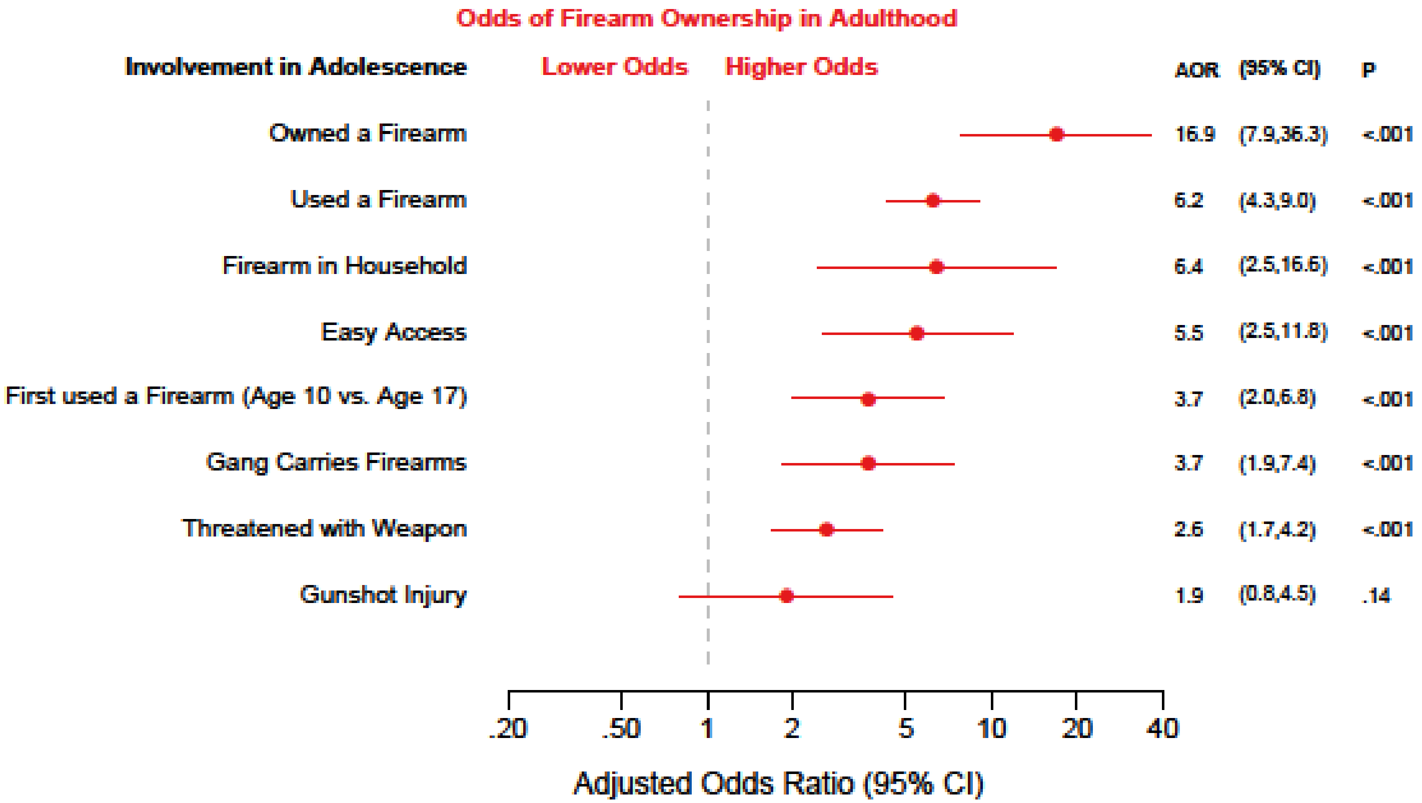

© 2021 Teplin LA et al. *JAMA Network Open.*

eReferences

1. Snyder HN, Sickmund M. *Juvenile Offenders and Victims: 2006 National Report.* Washington, DC: U.S. Department of Justice, Office of Justice Programs, Office of Juvenile Justice and Delinquency Prevention;2006.

2. Teplin LA, Abram KM, McClelland GM, Dulcan MK, Mericle AA. Psychiatric disorders in youth in juvenile detention. *Arch Gen Psychiatry.* 2002;59(12):1133-1143.

3. Puzzanchera C, Stahl A, Finnegan TA, Tierney N, Snyder HN. *Juvenile Court Statistics 1999.* Pittsburg PA: Office of Juvenile Justice and Delinquency Prevention;2003. NCJ # 201241.

4. General Accounting Office. *Juvenile Justice: Juveniles Processed in Criminal Court and Case Dispositions.* Washington, DC: US General Accounting Office;1995. GAO/GGD-95-170.

5. Griffin P. *Trying and sentencing juveniles as adults: an analysis of state transfer and blended sentencing laws. Technical Assistance to the Juvenile Court: Special Project Bulletin.* Pittsburg, PA: National Center for Juvenile Justice;2003.

6. Snyder HN, Sickmund M, Poe-Yamagata E. *Juvenile Transfers to Criminal Court in the 1990's: Lessons Learned From Four Studies.* Washington, DC: US Department of Justice, Office of Juvenile Justice and Delinquency Prevention;2000.

7. Salekin RT, Yff R, Neumann CS, Leistico A-MR, Zalot AA. Juvenile transfer to adult courts: A look at the prototypes for dangerousness sophistication-maturity and amenability to treatment through a legal lens. *Psychology, Public Policy, and Law.* 2002;8(4):373-410.

8. Austin J, Johnson KD, Gregoriou M. *Juveniles in Adult Prisons and Jails: A National Assessment.* Washington, DC: Office of Juvenile Justice and Delinquency Prevention;2000.

9. Washburn JJ, Teplin LA, Voss LS, Simon CD, Abram KM, McClelland GM. Psychiatric Disorders Among Detained Youths: A Comparison of Youths Processed in Juvenile Court and Adult Criminal Court. *Psychiatric Services.* 2008;59(9):965-973.

10. Federal Policy for the Protection of Human Subjects: Notices and Rules: Part 2. *Federal Register.* 1991;56(117):28002-28032.

11. Fisher CB. Integrating science and ethics in research with high-risk children and youth. *Social Policy Report.* 1993;7(4):1-27.

12. Institute of Behavioral Science. *Denver Youth Survey Youth Interview Schedule.* Boulder: University of Colorado; 1991.

13. Harrell FE, Jr. *Regression modeling strategies: With applications to linear models.* New York, NY: Spring; 2001.

14. Durrleman S, Simon R. Flexible regression models with cubic splines. *Stat Med.* 1989;8(5):551-561.

15. Gauthier J, Wu QV, Gooley TA. Cubic splines to model relationships between continuous variables and outcomes: a guide for clinicians. *Bone Marrow Transplantation.* 2020;55(4):675-680.

© 2021 Teplin LA et al. *JAMA Network Open.*
